# Healthy snacks at the checkout counter: A lab and field study on the impact of shelf arrangement and assortment structure on consumer choices

**DOI:** 10.1186/1471-2458-12-1072

**Published:** 2012-12-12

**Authors:** Ellen van Kleef, Kai Otten, Hans CM van Trijp

**Affiliations:** 1 Wageningen University, Marketing and Consumer Behaviour Group, Hollandseweg 1, Wageningen, The Netherlands

**Keywords:** Nudging, Obesity prevention, Environmental interventions, Snacking, Food accessibility, Food availability, Choice architecture

## Abstract

**Background:**

The essence of nudging is to adapt the environment in which consumers make decisions to help them make better choices, without forcing certain outcomes upon them. To determine how consumers can effectively be guided to select healthier snacks, we examine the effect of manipulating the assortment structure and shelf layout of an impulse display including both healthy and unhealthy snacks near the checkout counter of a canteen.

**Methods:**

Both a lab and field study applied a two-factor experimental design manipulating snack offerings both in an on-screen choice environment and a natural environment (hospital staff restaurant). Shelf arrangement (i.e. accessibility) was altered by putting healthy snacks at higher shelves versus lower shelves. Assortment structure (i.e. availability) was altered by offering an assortment that either included 25% or 75% healthy snacks. Participants in the lab study (n = 158) made a choice from a shelf display. A brief survey following snack selection asked participants to evaluate the assortment and their choice. The field experiment took place in a hospital canteen. Daily sales data were collected for a period of four weeks. On completion of the field study, employees (n = 92) filled out a questionnaire about all four displays and rated their attractiveness, healthiness and perceived freedom of choice.

**Results:**

The lab study showed a higher probability of healthy snack choice when 75% of the assortment consisted of healthy snacks compared to conditions with 25% healthy snack assortments, even though choices were not rated less satisfying or more restrictive. Regarding shelf display location of healthy snacks, no significant differences were observed. There was also no significant shelf arrangement by assortment structure interactive effect. The field study replicated these findings, in that this assortment structure led to higher sales of healthy snacks. Sales of unhealthy and total snacks were not impacted by manipulations (no main or interaction effects). Employees preferred shelf displays including a larger healthy snack assortment located at top shelves. Employees also felt more freedom in choice when healthy snacks were displayed at top shelves compared to lower shelves.

**Conclusions:**

Overall, results suggest that increasing the prominence of healthy snacks by enlarging their availability, while permitting access to unhealthy snacks, is a promising strategy to promote sales. These results point to the importance of nudging strategies to encourage healthier snack patterns.

## Background

Worldwide, overweight and obesity have become a major public health problem as they constitute a considerable risk for many chronic diseases such as certain cancers, type 2 diabetes and coronary heart diseases [1,2]. Consequently, effective public health strategies are urgently needed to prevent overweight and obesity and reduce the burden of disease [3]. A high intake of energy dense foods has been associated with excess weight gain [4]. Over the past decades, the number of snacking occasions per day and energy density of snacks have increased substantially [5].

As empirical evidence for the strong environmental influence on overeating and excess weight gain is growing [6], environmental approaches are increasingly being advocated as offering important potential for improving eating habits. Consumers make their decisions in an environment in which many cues may influence their purchase, although they are often not aware of this influence. Increasingly, efforts are being done to reshape the environment in which consumers make their food decisions [7]. Recently, the concept of nudging has become very popular in this respect [8]. Nudging works from the setting in which a choice is presented and aims to make beneficial choices (from a consumers’ perspective) more appealing [9]. They can be seen as relatively simple, easy to implement and inexpensive interventions. A key characteristic of nudging is that consumers maintain their liberty of choice [10]. This implies that interventions do not ban ‘forbidden’ products or enforce consumers to make a particular choice.

The present study examines the possibility of nudging consumers towards healthier snack choices near the checkout counter by altering the accessibility and availability of both healthy and unhealthy snacks. Both availability and easy accessibility of energy dense foods have been identified as risk factors for overeating [11]. In food studies, availability and accessibility has been conceptualized in various ways, such as spatial accessibility and means of transportation to stores [12]. Both of them have been recognized as promising starting points to design effective intervention tools. Interventions based on these features have typically been studied separately or combined with other interventions such as price alterations or labelling. For example, results from an intervention at a large hospital cafeteria suggest that a color-coded labelling intervention in combination with a rearrangement of healthy items (displaying them at eye level) can contribute to increased sales of these items [13]. In another study, accessibility of (un)healthy foods and the provision of caloric information were manipulated on paper menus distributed at a fast food sandwich chain. It was found that participants were more likely to choose low-calorie options when these were put front-page rather than presented at the back page of the menu [14]. Hanks and colleagues [15] showed that adding a convenience line displaying only healthy foods in a school lunch room increased sales of healthier food by 18% and decreased sales of less healthy foods by nearly 28%. Maas and colleagues [12] manipulated accessibility by changing distance to unhealthy snacks in a lab study. They found that putting snacks further away reduced the likelihood and amount of snack intake. Similarly, making a food slightly more difficult to reach reduced intake of salad bar food by 8-16% [16]. Not all studies find such strong effects, probably because of strong existing preferences for particular products [17]. For example, Meyers and colleagues [18] manipulated the accessibility of high- and low-calorie desserts in a hospital cafeteria. Making low-calorie desserts less accessible decreased the likelihood of their selection and resulted in fewer desserts taken at all. However, when high-calorie desserts were made less accessible and visually salient, people accepted the inconvenience of reaching for them rather than eating a low calorie dessert. In sum, the few experimental studies that have explored accessibility provide promising but sometimes inconclusive results, probably due to different type of manipulations and settings (i.e. lab environment, school, hospital canteen, fast food chain).

Besides manipulating accessibility or convenience, studies manipulating availability typically origin in the marketing field [19,20]. For example, although this study was conducted almost four decades ago, Curhan [20] showed that doubling shelf space for hard fruit in a supermarket increased sales by 44%. In a more recent study on vending machines in bus garages, the number of available healthy items was increased while at the same time prices of these items were lowered. Although the effect of lowering prices cannot be separated from the effect of increasing availability, the interventions resulted in 10-42% higher sales of healthy items [21]. All in all, previous studies particularly focused on the effects of accessibility, while availability has received less research attention in the field of public health promotion. In this study, accessibility is defined as the convenience or closeness of physically obtaining a product in a shelf space. We define availability as the presence of snacks ready for immediate choice by consumers.

No research to date combined both the effects of availability and accessibility to better understand their potential as a simple strategy to help people make healthier food choices in a worksite canteen. To inform new policy making in public health, the contribution of this paper is that we address the effect of availability on healthy food choices, both as a main effect and in combination with accessibility that has been studied more widely. Moreover, a lab study alone is not robust enough to provide reliable predictions about how people behave in a real-life context. Therefore, we address these effects both at the fundamental level (controlled lab experiment) as well as at the public health implication level (a realistic real life setting).

Research in the field of behavioural economics has shown that consumer decisions are often irrational and prone to biases [10]. Visitors of a self-service restaurant or canteen are often in a hurry and tend to be hungry, which makes them more prone to these biases. The interventions in our study aim to take advantage of biases related to easy accessibility and availability. Altering the ease of access to snacks basically means that it requires less effort to obtain them. People furthermore have the tendency to go for the default option as this typically requires the least effort due to habits or even ‘laziness’ [22]. Availability can influence consumer choices in various ways. More healthy snacks present for purchase increases the likelihood that consumers find a snack that fits their need. Higher availability of healthy snacks leads to a larger assortment which tend to raise consumer expectations and satisfy consumers with a high need for variety [19,23]. By enlarging the available assortment of healthy snacks, we made these snacks the implicit default. This may function as a cue that implies a consumption norm. Typical checkout counter assortments including a variety of chocolate bars, candy and savoury snacks speak to the desire of immediate pleasure rather than uncertain rewards such as achieving a healthy body weight. Food products and snack displays are often designed to stimulate the automatic affective system of human behaviour, which is being driven by triggers in the environment. Therefore, our intervention aims to make taking a healthy snack slightly more convenient and attractive while preserving freedom of choice.

Recently, ethical concerns have been raised that nudges could be too intrusive and restrict (perceived) freedom of choice [24,25]. For example, strategies that focus on offering people a default option are problematic if people tend to overlook the possibility to make alternative choices [26]. Moreover, according to reactance theory [27], eliminating choice options or putting pressure on people to eat healthy may lead to reactance in that some consumers will do exactly the opposite (e.g. go for the unhealthy option). Therefore, an additional aim of this study is that we also examined participants’ perception of their freedom in making choices and choice satisfaction.

The first study reported provides a lab experiment into whether shelf arrangement (location of healthy snacks on top versus bottom of shelf display) and assortment structure (assortment includes either 75% or 25% healthy snacks) influences the type of choices consumers make. Pictures of shelf displays were created to simulate snack food displays that can typically be found near the checkout counter of self-service restaurants or canteens. Because this study was conducted with tightly controlled stimulus material in an imaginative choice situation, the question is whether the results would generalize to a ‘real-world’ snack choice situation. Therefore, the second study is a field experiment in which the same manipulations were applied to an actual snack shelf display in a hospital canteen. Worksite restaurants are important intervention sites because of trend towards increasing out-of-home consumptions [28].

To summarize, changes in a snack assortment in terms of effort required to actually grab a product and the size of an assortment may make healthier snacks more salient, attractive, normative and convenient. For both studies, it was hypothesized that the individual and combined effect of increasing the availability of healthy snacks (while at the same time not banning unhealthy snacks) and positioning them at the top of the shelf display would increase sales of these healthy options. We manipulate the number of facings of healthy snacks and the vertical position of 16 snacks, while keeping total shelf space constant and retaining the availability of unhealthy options.

In sum, the objective of this article is to examine the effects and interplay between shelf arrangement and assortment structure on consumer choices for healthy and unhealthy snack products.

## Methods

### Methods lab study

#### Participants

Participants were 158 undergraduate students (103 women, 55 men) who were invited by e-mail to complete an online questionnaire in exchange for a chance to win a gift certificate. Participants gave their informed consent before taking part in the study and completed the questionnaire on their own computer at any time they wanted. Participants’ mean age was 21.8 (SD = 6.7). Both studies (lab and field study) were approved by the Social Sciences Ethical Committee of Wageningen University.

#### Procedure and materials

**Figure 1:**
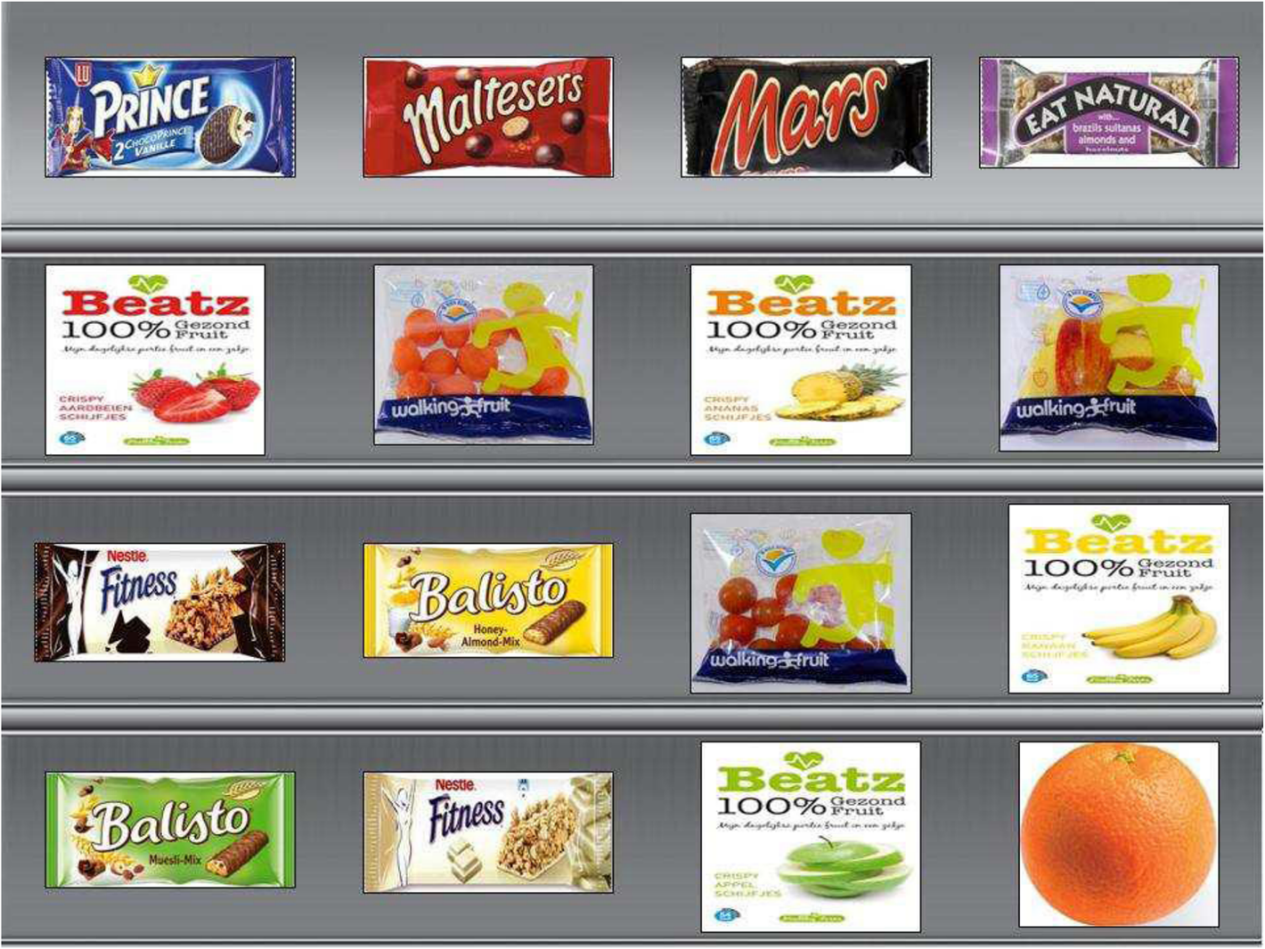
Screenshot of one of the virtual shelves (75% healthy, on bottom).

Participants were randomly assigned to one of four conditions in a 2 (shelf arrangement: healthy snacks on top versus bottom shelves) by 2 (assortment structure: 75% healthy snacks in assortment versus 25%) between-subject design. Participants were shown a picture of a shelf display with photographs of single-portion snack products (Figure 1). Each shelf display contained 16 photographs of snack products. All photographs were of the same size and shot from the same angle. Snack options were organized in a four row ‘table’ with four snacks appearing in each row. Across conditions, there were always 16 available snack options. All participants were shown both healthy and unhealthy snacks, but how many of each appeared (4 or 12) varied, as well as the position of either healthy or unhealthy snacks (top or bottom). Participants were asked to imagine that they were at the university canteen around 4 o’clock in the afternoon. After seeing the shelf display, they were asked to select their favourite snack by clicking on the product. 

A total of 24 snacks were selected for inclusion in the experiment: all were available in Dutch supermarkets. Selection of 12 relatively healthy and 12 unhealthy snacks was based on guidelines from the Netherlands Nutrition Centre (governmental funded organization responsible for public nutrition education [29]). Their guidelines are based on a report of the Dutch Health Council [30] and categorize snack foods into three groups (preferred, by exception and ‘in-between’). For this study, snacks were considered healthy if they would fit the preferred or ‘in- between’ category and hence contained less than 110 calories per portion. We selected a variety of snacks from the product groups: (1) fresh and dried fruit and vegetables, (2) savoury and salty snacks, and (3) sweet biscuits and chocolates. Examples of single-portion healthy items included: fresh fruit (banana, apple, orange), dried fruit chips, snack-sized cucumbers and tomatoes, and Balisto granola bar. Examples of unhealthy snacks included single-portion cookies, sausage, chocolate bars (e.g. Mars), rice chips and candy pouches. Across conditions, participants could always select a fresh fruit or vegetable, dried fruit, savoury snack or biscuit.

#### Measures

The key dependent variable was participants’ choice (after the shelf display manipulation). After participants made their snack choice, they completed a brief questionnaire. The extent to which participants were satisfied with their choice was measured on a 3-item scale (i.e., ‘I am satisfied with my choice’, ‘the snack I selected seems tasty’, and ‘I made a good choice’). The reliability of this scale was α = 0.73. To rule out reasons why the manipulations may (not) influence choice, assortment realism and mood were measured as manipulation checks. Realism of the assortment was measured by two items: ‘the display looks realistic’ and ‘I would expect such a display near the checkout counter’. The reliability of this scale was α = 0.70. All items were answered on a seven-point scale, with anchors of 1 (strongly disagree) and 7 (strongly agree). Participants were also asked how they felt when choosing their snack. Two dimensions of mood were measured with bipolar items (good mood-bad mood; relaxed-agitated).

Perceived freedom of product choice was measured by three bipolar adjectives (influenced by the situation-not influenced by the situation; not free in making a choice-free in making a choice; steered-unrestricted). Scores on these items were averaged (α = 0.79). Each bipolar item was measured on a seven-point scale. Finally, we measured how hungry participants felt on a 7-point scale (1 = not at all hungry, 7 = very hungry).

#### Data analysis

The main outcome variable in this study is the snack choice made by participants. Logistic regression analysis was conducted with healthy snack choice as key dependent variable, coded as 1 if participants selected a healthy snack and 0 if they selected an unhealthy snack. Healthy snack choice was regressed on shelf arrangement, assortment structure and their interaction. Ratings of snack choice satisfaction, assortment realism, mood and perceived freedom of product choice were analysed using analyses of variance (ANOVA) with these ratings as dependent variables and shelf arrangement and assortment structure as independent variables. All analyses were performed using SPSS statistical software (SPSS version 19.0.0.1 SPSS Inc., Chicago, IL, 2011).

### Methods field study

#### Design and procedures

**Figure 2:**
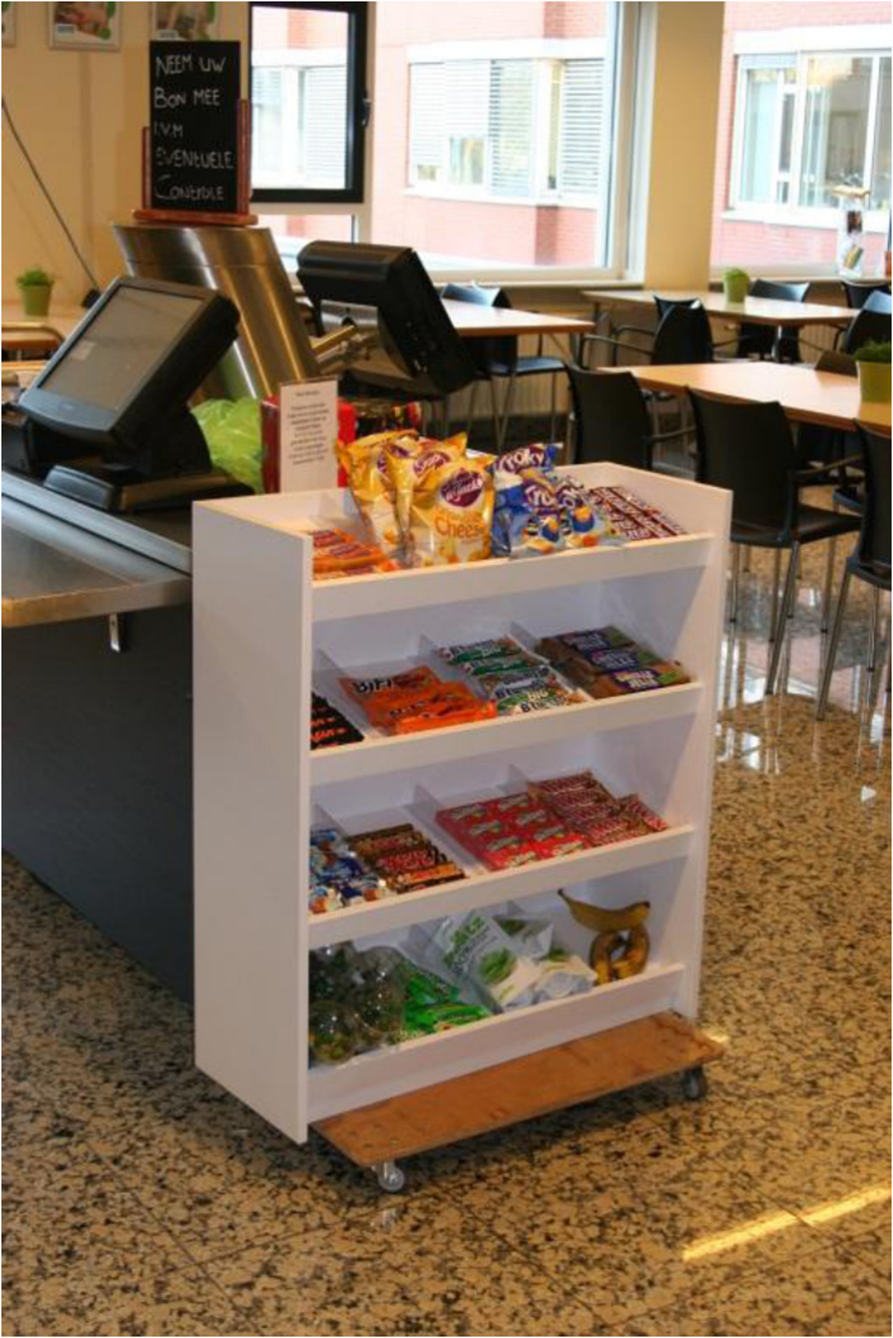
Shelf display in front of checkout counters.

The field study employed a similar two factor experimental design of assortment structure and shelf arrangement, but now snacks were displayed in an actual shelf with snacks placed at the staff canteen of a middle sized Dutch hospital canteen. As this study was conducted in the field rather than the laboratory, no participants could be randomly assigned to each condition. Therefore, an experimental design was employed in which four successive weeks were randomly assigned to the four experimental conditions. So, each week an alternative snack arrangement was on display. A specially constructed wooden snack product display (height = 89 centimetres, length = 79 centimetres, depth = 30 centimetres) was placed in front of the check-out counter (see Figure 2). Data collection took place during October and November 2011. Although the same nutrition criteria to select healthy and unhealthy snacks were applied, due to availability not all type of snacks were similar to the snacks used in the lab study. The number of available snacks was the same: 16 in total (12 versus 4 depending on conditions). 

Although the lunch buffet is served daily from 11.30 am to 1.30 pm, visitors are allowed to purchase displayed snacks at a self-service checkout counter throughout the day. About 500 people per weekday purchased items in the cafeteria (less during weekend days). All products were sold at €0.85 except for fresh fruits (i.e., apples, oranges and bananas) which were sold at €0.50. All four conditions of assortment structures were displayed for one week (including weekend days). One week prior to the experiment, the shelf display filled with a variety of snacks was put near the checkout counter to get visitors familiar with its appearance. Prior to that, only a very limited number of snacks were sold by the staff canteen. During the four experimental weeks, sales data were collected by staff canteen employees on a daily basis by counting the number of snacks left at the end of each day. It was not communicated to visitors that a study was going on. The collected data consisted of the total number of snacks sold, the number of individual snack products sold and the number of visitors for that day. The shelf displays were weekly changed by a member of the research team at the same time (Monday morning around 9 am). Staff canteen employees were instructed to frequently restock the shelf displays to ensure that no empty shelves would be visible.

#### Survey among hospital cafeteria visitors

Starting in the week after completion of the intervention, all hospital employees visiting the staff canteen were offered the opportunity to fill in a questionnaire. The questionnaire was announced at the hospital’s intranet as a survey being carried out by a Dutch university in which visitors could express their opinion about the staff canteen. No reference was made to the intervention that had been taken place. Potential participants were also approached in the hospital canteen. Those who agreed to participate were asked to provide their e-mail address at which they subsequently received an invitation with link to the online questionnaire. In total, 92 employees (82 women, 10 men) of the about 2900 employees working in the hospital completed the questionnaire and gave informed consent.

The questionnaire started with an open-ended question about respondents’ general opinion of the canteen assortment. Next, to determine whether visitors had been aware of the intervention, participants were asked whether they had noticed assortment changes during the previous months. If confirmed, an open-ended question asked for a brief explanation of the noticed changes. The next task consisted of a sequential evaluation of photographs of the four different shelf displays. The photographs were taken at the staff canteen from the same angle. Shelf display attractiveness was measured by three items (i.e. ‘this shelf display offers novel choice options’, ‘choosing from this shelf display is simple’ and ‘this is a shelf display packed with attractive snacks’). The reliability of this scale was α = 0.77. Shelf display healthiness was measured by two items: ‘this is a shelf display that makes a healthy choice easy’ and ‘this is a shelf display packed with healthy snacks’ (α = 0.87). All items were answered on a seven-point scale, with anchors of 1 (strongly disagree) and 7 (strongly agree). Perceived freedom in choice was similarly measured as in the lab study (α = 0.73 in this study). The order of the four shelf displays was randomized across participants. Finally, participants were asked to indicate their favourite shelf display by clicking on one of the four photographs showing the four displays.

#### Data analysis

Daily field study sales data was analysed using a series of repeated measures ANOVA with both assortment structure and shelf arrangement as within subjects factors (two levels each). The dependent variables in the analysis are the sales of all snacks, the sales of all healthy snacks and the sales of all unhealthy snacks. To account for the number of paying guests in the staff canteen, all daily sales data were divided by the number of visitors of that particular day.

Regarding the survey among employees of the hospital, a repeated measures ANOVA with both assortment structure and shelf arrangement as within subjects factors was used to assess differences in shelf display evaluations (attractiveness, healthiness and perceived freedom in product choice). Differences in means were considered significant at p < .05.

## Results

### Results lab study

Overall, 46 out of 158 participants chose a healthy snack and the remaining 112 participants selected an unhealthy snack. There were no differences in gender (*χ*^2^(1, N = 158) = 0.03, p = .87) or feelings of hunger (all Fs < 1.70, all ps > 0.19) across the four conditions. However, there was a trend towards an age difference across conditions (interaction between shelf arrangement and assortment structure: F(1,154) = 3.71, p = .06) and therefore in the logistic regression analysis we included age as covariate to control for influence.

**Figure 3:**
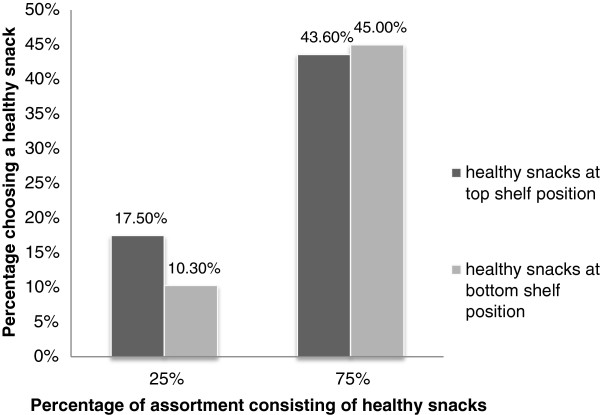
Choices made by participants from online shelf displays (lab study).

Logistic regression showed a main effect of assortment structure on healthy snack choice (*χ2*(*1*, *N* = *158*) = 10.84, *p* =0.001). Across both conditions that included an assortment with 75% healthy snacks, 44.3% selected a healthy snack, versus 13.9% of all participants exposed to conditions which included 25% healthy snacks (Figure 3). The odds ratio was 0.34 with a 95% confidence interval of 0.18 to 0.65. The inverted odds ratio showed that participants exposed to a shelf with 75% of the assortment consisting of healthy snacks are 2.9 times more likely to select a healthy snack than consumers who are exposed to a shelf with 25% of the assortment being healthy. Regarding shelf display location of healthy snacks, no significant differences were observed in the ‘healthy snacks at the top’ conditions (30.38% choose healthy) compared to the bottom conditions (27.85%; *χ2*(*1*, *N* = *158*) = 1.29, p = 0.34). There was also no significant shelf arrangement by assortment structure interactive effect on healthy snack choice (*χ2*(*1*, *N* = *158*) = 1.12, *p* =0.29). 

ANOVAs with shelf arrangement and assortment structure as independent variables and ratings of snack choice satisfaction, perceived freedom of choice and mood state during choice task as dependent variables revealed no significant main or interaction effects (all Fs ≤ 3.33, see Table 1). A similar ANOVA with ‘assortment realism’ as dependent variable, however, revealed a main effect of assortment structure in that participants in conditions where 75% of the assortment consisted out of healthy snacks rated the assortment as less realistic than participants in 25% healthy snacks assortment (75% assortment: M = 4.1, SD = 1.4; 25% assortment: M = 4.7, SD = 1.4). There was no main effect of shelf display or interaction effects of shelf display and assortment structure on this variable (both Fs ≤ 1.81). 

**Table 1:** Ratings of mood state, snack choice satisfaction, assortment realism and perceived freedom of product choice (mean, SD) in four shelf display conditions (lab study)

	**75% healthy snacks**		**25% healthy snacks**		**ANOVA (F)**		
**Dependent variable**	**Top (n = 39)**	**Bottom (n = 40)**	**Top (n = 40)**	**Bottom (n = 39)**	**Assortment structure (AT)**	**Shelf arrangement (SA)**	AT * SA
Mood state*							
Bad mood	2.8 (1.0)	2.8 (1.2)	2.7 (1.3)	2.9 (1.2)	0.01	0.12	0.10
Agitated	3.0 (1.3)	2.7 (1.6)	2.7 (1.3)	2.7 (1.6)	0.45	0.45	0.56
Snack choice satisfaction*	5.6 (1.3)	6.0 (0.7)	6.0 (0.7)	5.9 (0.6)	1.49	1.96	3.33
Assortment realism*	3.9 (1.6)	4.2 (1.2)	4.9 (1.3)	4.5 (1.5)	8.32^a^	0.06	1.81
Perceived freedom of product choice*	5.2 (1.2)	5.2 (1.6)	5.4 (1.3)	5.4 (1.4)	0.71	0.01	0.04

*measured at seven-*point* (*bipolar*) *scales*; ^*a*^ P = *0*.*01*.

### Results field study

#### Snacks sold

In total, 291 snacks were sold during the four-week period (200 healthy snacks and 91 unhealthy snacks). Repeated measures ANOVA showed no significant effects of assortment structure (F(1,6) = 0.19, p = 0.68), shelf arrangement (F(1,6) = 3.84, p = 0.10) or interaction (F(1,6) = 1.58, p = 0.26) on total snack sales. A separate repeated measures ANOVA on sales of unhealthy snacks showed similarly no main effect of assortment structure (F(1,6) = 3.99, p = 0.09), shelf arrangement (F(1,6) = 0.11. p = 0.75) or interaction between these two factors (F(1,6) = 1.17, p = 0.32), indicating that the snack display manipulations did not impact sales of unhealthy snacks.

**Figure 4:**
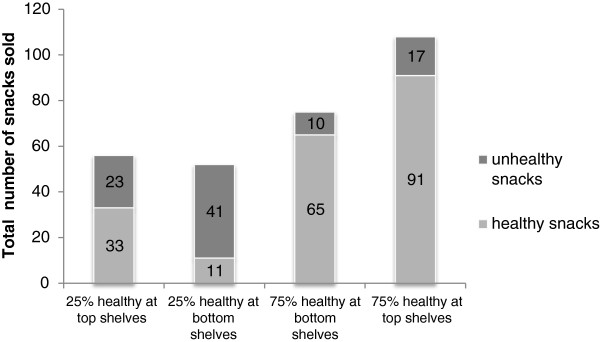
Total number of snacks sold in four conditions (field study).

For healthy snacks, however, a significant main effect of assortment structure was observed on sales (F(1,6) = 12.44, p = 0.01). As shown in Figure 4, more daily sales of healthy snacks (M = 11.1, SD = 6.8) were observed when 75% of the snacks were healthy compared to when 25% of the snacks were healthy (M = 3.1, SD = 2.3). In this latter analysis, there was no main effect of shelf arrangement (F(1,6) = 5.03, p = 0.07) although there is a trend towards an effect in the hypothesized direction. Also, no interaction effect of shelf arrangement and assortment structure was observed (F(1,6) = 1.03, p = 0.35). 

#### Survey

In the total sample, the mean age of participants was 41.3 years (*SD* = 11.3, age range was 16–63). Of all 92 participants, 57 (62%) indicated that they did not see any changes in the assortment or indicated changes that were unrelated to the intervention (e.g. ‘more variety in bread options’). The remaining 35 participants (38%) indicated assortment changes that could be related to the intervention, ranging from specific answers such as ‘a snack display near the checkout counter’ to imprecise answers such as ‘a broader assortment’.

Repeated measures ANOVA showed both a main effect of assortment structure (F(1,91) = 15.50, p < 0.001) and shelf arrangement (F(1,91) = 11.00, p < .001) on shelf display attractiveness ratings (Table 2). Looking at the mean ratings, this indicates that the more visible the healthy snacks are in terms of location (i.e. on top shelves) and proportion of assortment (i.e. 75% is healthy), the more attractive the entire assortment is perceived to be. No interaction effect occurred between shelf arrangement and assortment structure (F(1,91 = 1.84, p = 0.18). For shelf display healthiness ratings, repeated measures ANOVA showed both a main effect of assortment structure (F(1,91) = 114.03, p < .001) and shelf arrangement (F(1,91) = 36.22, p < .001). No interaction effect occurred between shelf arrangement and assortment structure (F(1,91) = 0.13, p = 0.72). 

**Table 2:** Shelf display preferences (% of participants indicating favourite display), attractiveness, healthiness and perceived freedom in product choice (mean, SD) for four shelf display conditions (survey field study, n = 92)

	**75% healthy snacks**		**25% healthy snacks**		**ANOVA (F)**		
**Variable**	**Top**	**Bottom**	**Top**	**Bottom**	**Assortment structure (AT)**	**Shelf arrangement (SA)**	AT * SA
Shelf display attractiveness*	4.9 (1.2)	4.7 (1.2)	4.5 (1.3)	4.1 (1.4)	15.50 ^*a*^	11.00 ^*a*^	1.84
Shelf display healthiness*	5.1 (1.3)	4.2 (1.5)	3.5 (1.6)	2.8 (1.4)	114.03 ^*a*^	36.22 ^*a*^	0.13
Perceived freedom in product choice*	4.8 (1.3)	4.6 (1.3)	4.7 (1.5)	4.5 (1.5)	1.23	6.63 ^*b*^	0.01
Preferred shelf display (% of total participants)	71%	4%	21%	4%	^-^	-	-

*measured at seven-point scales; ^*a*^*P* ≤ *0*.*001*, ^*b*^*P* ≤ *0*.*05*.

A separate repeated measures ANOVA on perceived freedom of choice ratings showed a main effect of shelf arrangement (F(1,91) = 6.63, p < 0.05), but no main effect of assortment structure (F(1,91) = 1.23, p = 0.27). No interaction effect occurred between shelf arrangement and assortment structure (F(1,91) = 0.01, p = 0.91). This indicates that healthy snacks positioned on top of the shelves obtained higher ratings of perceived freedom in choice, compared to displays where these healthy snacks are located at lower shelves. Finally, we asked participants to indicate their preferred shelf display. A majority of the participants (71%) selected the shelf display with 75% healthy snacks located on top.

## Discussions and conclusions

In this paper, we presented two experiments, one controlled lab study and one field study in a hospital canteen that examine the combined effects of the availability and shelf arrangement of healthy snacks on snack decisions and consumer perceptions of assortment and choice. The results of both studies show that changing the snack assortment can change consumers’ choice in a more healthful direction when the majority of the assortment consists of healthy options while allowing for more unhealthy choices. Within the setting that we investigated, more shelf space for healthy snacks increased the likelihood that people choose for a healthy snack (lab study) and led to higher sales of healthy snacks (field study).

There are several possible explanations for these findings. A probable mechanism which could explain our findings is that people try to minimize the energy spent in obtaining food [16]. More healthy foods in shelves probably enhanced their desirability and visibility. Normally, the small number of fruit and other healthy snacks hardly attract consumers’ attention. Measurement of shelf spaces in various types of stores showed that stores all devote more shelf space to unhealthy than healthy items. Smaller shops such as convenience shops typically assign a disproportionally high amount of shelf space to more unhealthy food such as energy rich snacks foods and beverages [31]. As such, our findings resemble findings of marketing studies showing that the number of shelf facings has strong impact on how consumers pay attention and evaluate products and brands [32,33]. A larger assortment also makes it more likely that consumers find a product that fits their needs. Recall that across studies, we manipulated both the proportion of available healthy snacks and the position of these snacks at shelves. As a result, not only the number of available products, but also the product variety within type of snack (i.e. healthy or unhealthy) differed across conditions. Previous research suggests that more variety increases satisfaction [34]. Unfortunately, our design did not allow for disentangling this combined effect of number and variety of options. Future research could address this issue.

Putting healthy snacks at the most convenient (top) shelf space did not impact consumer choices and sales, as both the lab study and the field study showed. In other words, making unhealthy snacks less accessible did not discourage their selection. Although a marginal effect was obtained for shelf arrangement in that snacks on top shelves were more often selected, this effect did not reach statistical significance. This absence of an effect of accessibility was unexpected. It may be that the accessibility manipulation was not strong enough to actually change convenience or the amount of data was too limited. The simulated choice task in the lab study probably did not change the nearness of item selection. In the field study, visitors had to bend at their knees to obtain a snack from the lower shelves. Furthermore, a limitation of adding healthy alternatives to choice sets can be that this increases the consumption of indulgent food items [35]. In that case, the presence of healthy items provides a license to indulge. We could not find such an effect.

Nudging is based on the premise that it is justifiable to encourage consumers to make better choices, by gently pushing them in the right direction. Although the majority of respondents of the field study survey did not observe any changes in the assortment, for a group of employees the special and new shelf display captured their attention. Many choices in settings such as canteens and grocery stores are relatively low involvement choices; consumers do not actively process available information about choice alternatives. However, the unexpected display in the canteen may have led to more effortful attention of employees, who might otherwise neglect it after a longer time period. Interestingly, although our manipulations did not alter perceptions of freedom of choice in the lab study, surveyed hospital employees experienced more freedom in snack choice in situations were healthy snacks were visibly at top shelves. This may be understandable given their strong preference for a relatively large healthy snack assortment. Overall, about 31% of all sold snacks in the hospital were unhealthy while for students in the labs study about 71% of all snacks choices were unhealthy. This could be the result of the different samples studied, each with its own snack preferences. It could also be related to expectations that people have of the type of snacks that are appropriate to eat within the context that they buy them (hospital versus university canteen). Additionally, the assortments with 75% unhealthy snacks were seen as more realistic than assortments with 25% unhealthy snacks. The increased healthy choices may be due to the demand effects of the research context which may have hinted at health and food choices.

Despite the clear results, some limitations must be noted about both studies. One is the artificial nature of the choice task in the first lab study and the involvement of student participants. Although the assortments in the lab study including 75% healthy snacks were seen as less realistic, results showed that our manipulation did not alter choice satisfaction. Perhaps the assortment did not look like a typical snack assortment at a university canteen or the instruction being given (select favourite snack) influenced choice. Another limitation refers to the relative small number of snacks sold. In the field study, fruit had a lower price which may have given these products an additional benefit. Moreover, it is unclear whether our intervention could produce sustained changes in snack choice behaviour. It may be possible that eating a healthy snack is compensated later in the day by eating or snacking more. Despite such limitations, our experiments demonstrated that increasing the availability of healthy snacks may be effective in a canteen setting. Caution, however, should be exercised in generalizing these findings to other settings such as grocery stores or restaurants. Seymour and colleagues [36] reviewed the effectiveness of nutritional interventions in worksite and university cafeterias involving the availability, access to, pricing or and information about fruit and vegetables. They concluded that these interventions are more likely to be successful than those in restaurant and grocery stores. This is because these ‘limited access’ sites have fewer other options available. Nevertheless, the work environment can have a large impact on food choices as people tend to consume a large part of their daily energy intake during their work day. Moreover, previous research has shown that a healthy worksite food environment can have productivity-enhancing effects [37].

Our results show that a relative large assortment of healthy snacks is able to influence consumers’ healthy snack purchases. With the present studies, we extend our understanding of the effects of accessibility and availability of snack foods on consumer choices. Our research examined two factors that might play a role in healthy snack choices. An interesting topic for further research would involve the identification of other important factors that gently nudge consumers towards healthier snack choices and the effectiveness of these factors in various contexts.

## Competing interests

The authors declare that they have not competing interests.

## Authors’ contributions

All authors (EvK, KO, and HvT) contributed to the design of the study. EvK and KO conducted the statistical analysis and drafted the manuscript. All authors participated in the interpretation of the data and critical revision of the manuscript for important intellectual content. All authors read and approved the final manuscript.

## Pre-publication history

The pre-publication history for this paper can be accessed here:

http://www.biomedcentral.com/1471-2458/12/1072/prepub
